# Inclusion body myositis and Sjögren’s syndrome: the association works both ways

**DOI:** 10.1186/s40478-022-01443-3

**Published:** 2022-10-24

**Authors:** Margherita Giannini, Renaud Felten, Jacques-Eric Gottenberg, Bernard Geny, Alain Meyer

**Affiliations:** 1 Exploration fonctionnelle musculaire, Service de Physiologie Exploration Fonctionnelle, CRBS Hôpitaux Universitaires de Strasbourg Université de Strasbourg, EA 3072 Strasbourg, France; 2 Service de rhumatologie, Centre de Référence des Maladies Auto-immunes Systémiques Rares Est Sud-Ouest Hôpitaux Universitaires de Strasbourg, Université de Strasbourg, Strasbourg, France

**Keywords:** Myositis, Inflammatory myopathies, Dermatomyositis, Polymyositis, Antisynthetase syndrome, Inclusion body myositis, Sjögren’s syndrome

Dear Editor,

We read with great interest the article by Nelke et al. [1] reviewing the situations where inclusion body myositis (IBM) occurs in the context of other diseases, especially Sjögren’s syndrome (SjS) and would like to additionally point out data that recently shed new light on this association.

Firstly, based on two previous (2002 and 2015) retrospective cross sectional cohorts that used non-consensual criteria for both SjS and IBM diagnosis [2, 3], Nelke et al. reported that the prevalence of IBM in SS is currently uncertain (between 0.0008 and 0.6%). However, studying a prospective national multicenter cohort of primary SjS (revised American-European criteria) systematically screened for muscle involvement during a 5 years follow-up, we recently reported a prevalence of IBM (Lloyd et al. criteria) of 0.5% [4] that was much higher than the prevalence in the general population we previously found in the meta-analysis of the available literature (2.01/100 000 [95% CI 1.51–2.69]) [5].

Secondly, discussing a study based on administrative data (ICD9 and ICD10), Nelke et al. reported a more likely association of SjS with IBM over the other subtypes of myositis [6]. Further demonstrating this point, in an independent monocentric myositis cohort (ACR/EULAR criteria), deeply characterized at the clinical, serological and histopathological levels, covering the entire myositis spectrum and systematically screened for SjS (ACR/EULAR criteria), we showed that myositis patients with SjS more frequently present IBM than myositis patients without SjS (24% vs. 6% *p* = 0.02) [7].

Finally, Nelke et al. suggested the association of IBM and SjS is characterized by anti-cN1A antibodies. Yet, we recently showed that in myositis patients, the association between anti-cN1A and SjS is independent of the association between IBM and SjS. Consequently, the specificity of anti-cN1A for IBM was excellent in myositis patients without SjS (0.96, 95% CI: 0.87, 0.99), but was limited in myositis patients with SjS (0.70, 95% CI: 0.48, 0.85) [7].

In conclusion, these recent data complete the nice review by Nelke et al. and increase the body of evidence that IBM and SjS are associated, raising caution about using the value of anti-cN1A for the diagnosis of IBM in SjS patients.
